# Efficacy and safety of liver support devices in acute and hyperacute liver failure: a systematic review and network meta-analysis

**DOI:** 10.1038/s41598-021-83292-z

**Published:** 2021-02-18

**Authors:** Anna Kanjo, Klementina Ocskay, Noémi Gede, Szabolcs Kiss, Zsolt Szakács, Andrea Párniczky, Steffen Mitzner, Jan Stange, Péter Hegyi, Zsolt Molnár

**Affiliations:** 1 Institute for Translational Medicine, Medical School, University of Pécs, Pécs, Hungary; 2 Heim Pál National Paediatric Institute, Budapest, Hungary; 3 Doctoral School of Clinical Medicine, University of Szeged, Szeged, Hungary; 4 János Szentágothai Research Centre, University of Pécs, Pécs, Hungary; 5 Division of Nephrology, Department of Medicine, University of Rostock, Rostock, Germany; 6 Department of Anesthesiology and Intensive Therapy, Poznan University for Medical Sciences, Medical Faculty, Poznan, Poland

**Keywords:** Hepatology, Gastroenterology

## Abstract

Acute liver failure (ALF) is a potentially life-threatening condition. Liver support therapies can be applied as a bridging-to-transplantation or bridging-to-recovery; however, results of clinical trials are controversial. Our aim was to compare liver support systems in acute and hyperacute liver failure with network meta-analysis. After systematic search, randomized controlled trials (RCT) comparing liver support therapies in adults with acute or hyperacute liver failure were included. In-hospital mortality was the primary outcome, the secondary outcomes were hepatic encephalopathy and mortality-by-aetiology. A Bayesian-method was used to perform network meta-analysis and calculate surface under the cumulative ranking curve (SUCRA) values to rank interventions. Eleven RCTs were included. BioLogic-DT and molecular adsorbent recirculating system (MARS) resulted in the lowest mortality (SUCRAs: 76% and 73%, respectively). In non-paracetamol-poisoned patients, BioLogic-DT, charcoal hemoperfusion and MARS may be equally efficient regarding mortality (SUCRAs: 53%, 52% and 52%, respectively). Considering hepatic encephalopathy, extracorporeal liver assist device (ELAD) may be the most effective option (SUCRA: 78%). However, in pairwise meta-analysis, there were no statistically significant differences between the interventions in the outcomes. In conclusion, MARS therapy seems to be the best available option in reducing mortality. Further research is needed on currently available and new therapeutic modalities. (CRD42020160133).

## Introduction

Acute and hyperacute liver failure are potentially life-threatening conditions that can lead to multiorgan failure^1,2^, affecting one and six per million people every year in developed countries^3^ with mortality rates of 25–50%^4–6^. The main causes of acute and hyperacute liver failure are drugs—especially paracetamol overdose (46–65%)—and viruses (29–77%), other etiologies are less frequent (11–23%) like mushroom poisoning, Budd-Chiari syndrome, Wilson-disease or HELLP-syndrome^6,7^. Due to the impaired synthetic and detoxification capacities, coagulopathy, jaundice and hepatic encephalopathy may develop^8^. In hyperacute liver failure considerably elevated transaminase levels and severe coagulopathy can be observed with slightly or not increased bilirubin levels^3^. Patients with hyperacute liver failure have a greater possibility to spontaneously recover without liver transplantation^3^.

Extracorporeal liver support systems (ECLS) can be used to aid the liver’s detoxification function by removing albumin-bound toxins and water-soluble substances^9^. Furthermore, bioartificial liver support therapies that contain hepatocytes can provide synthetic functions as well^10^. In liver failure when there is a potential for recovery, liver support systems amend the supportive care until the regeneration of the liver. In other cases, the definitive therapy of liver failure is liver transplantation—which is expensive and restricted by the number of organs available—however, liver support therapy can keep these patients alive until a suitable organ is found^11^. Considering the effectiveness of these therapies the results of clinical trials are controversial, thus, currently they are not recommended by thy European Association for the Study of the Liver (EASL) Clinical Practical Guidelines or the American Association for the Study of Liver Diseases (AASLD) Practice Guidelines outside of clinical trials in acute or hyperacute liver failure^12,13^.

In former meta-analyses in this field, the different interventions were considered equivalent and pooled together in comparison with standard medical therapy (SMT)^11,14–16^.

In conventional meta-analyses two interventions can be compared, however when multiple alternatives exist, network meta-analyses can provide results in a single analysis based on direct and indirect (no head-to-head trials conducted between the interventions before) comparisons as well^17^. Therefore, we decided to perform a network meta-analysis, in which we are able to assess the different liver support systems’ efficacy and safety in acute and hyperacute liver failure. With the statistical methods of network meta-analysis, we (1) compare the interventions to each other and (2) rank them, to choose the best option regarding the outcome.

## Results

### Selection process and study characteristics

**Figure 1:**
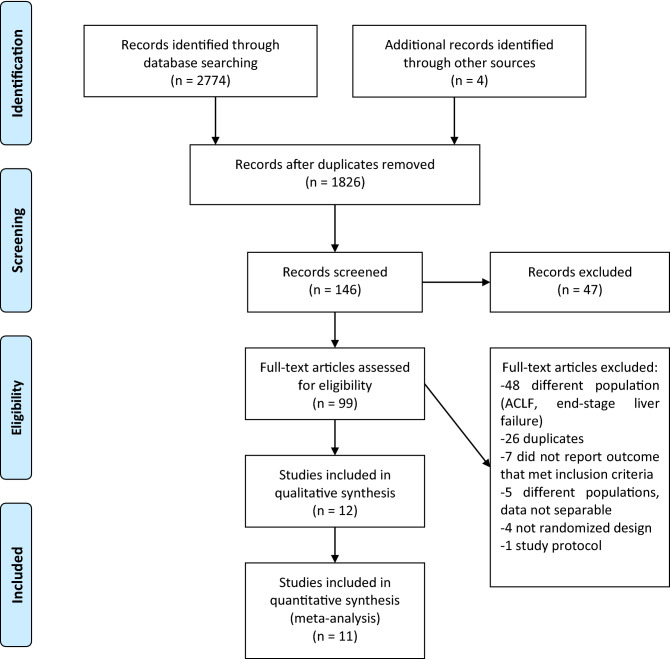
Study selection process. PRISMA flowchart containing results of systematic search and article selection. ACLF, acute-on-chronic liver failure.

Through the initial searches 2774 citations were identified. After reading the titles and abstracts, 99 articles remained for further assessment. 12 articles could be included for qualitative synthesis and 11 for network meta-analysis (Fig. 1). In the article of Demetriou et al., there were no data reported that we could include in the quantitative synthesis concerning mortality or hepatic encephalopathy^18^.

All studies included in the quantitative synthesis are parallel randomized controlled trials comparing liver support systems to SMT, published between 1973 and 2016, including 479 patients. Overall, 243 patients were assigned to a liver support therapy and 236 to SMT. In four of the studies BioLogic-DT^19–22^ (BioLogic-DT has been redesigned and now called Liver Dialysis Device^16^.), in three of them the Molecular Adsorbent Recirculating System (MARS) was applied^23–25^. Through the systematic search we found one study from each modalities analysing high-volume plasma exchange^26^, exchange transfusion^27^, Extracorporeal Liver Assist Device (ELAD)^28^ and charcoal hemoperfusion^29^. Bioartificial modalities are ELAD therapy (Vital Therapies Inc., San Diego, CA, USA) and HepatAssist device (Circe Biomedical Inc., Lexington, MA, USA). HepatAssist device was included only in the systematic review.

Seven studies reported detailed demographic characteristics. The mean age was 38.8 years, two studies included adolescents as well. About half of the sample population were female (55.8%—226 of 405). The majority of the studies included patients with different etiologies, however, the distribution of the different etiologic factors was similar to the general population. Seven RCTs recruited patients across Europe (58%), three in the USA (25%) and 2 multicentric trials recruited patients at the study sites across continents (17%) (Table 1).

**Table 1:** Randomized controlled trials included in the systematic review and network metaanalysis.

Study	Country	Population	Aetiology	Intervention (N^o^ of patients)	N^o^ of sessions	Ancillary hemodialysis (HD) and use of anticoagulant (AC) therapy	Comparator (N^o^ of patients)	Age range (mean)	Women (%)
Redeker (1973)	USA	ALF with gr. IV HE	Acute viral hepatitis (100%)	Exchange transfusion (n = 15)	Mean, SD: 1,1 ± 0.35, median: 1, range: 1–2, max: 2	AC: received	Standard medical therapy (n = 13)	16–67 (25.1)	39
O'Grady (1988)	UK	FHF with gr. IV HE	Acetaminophen overdose (AO) (52%), viral hepatitis (40%) drug reaction (8%)	Charcoal hemoperfusion (n = 29)	Median: 2, max: 4	HD: at the physician’s discretion AC: received	Standard medical therapy (n = 33)		
Hughes (1994)	UK	FHF with gr. IV HE	AO (60%), viral hepatitis (40%)	BioLogic-DT (n = 5)	Mean: 3.6, median: 4, range: 2–5, max: 5	HD: in case of renal failure, patients were excluded AC: not applied (producer’s suggestion)	Standard medical therapy (n = 5)	19–64 (37.3)	30
Ellis (1996)	UK	ALF	AO (71%), viral hepatitis (21%), drug induced (8%)	ELAD (n = 12)	Continuous	HD: at the physician’s discretion	Standard medical therapy (n = 12)	14–65	50
Mazariegos (1997)	USA	ALF with coma		BioLogic-DT (n = 5)	Max. 5		Standard medical therapy (n = 1)	35–65 (48.3)	67
Wilkinson (1998)	USA	ALF with gr. III-IV HE	Viral hepatitis (66%) heat stroke (33%)	BioLogic-DT (n = 1)	Mean: 3.6, max: 5	HD: in case of renal failure, patients were excluded AC: not applied (producer’s suggestion)	Standard medical therapy (n = 2)	27–58 (42.7)	33
Ellis (1999)	UK	ALF with gr. II or greater HE	Acute alcoholic hepatitis (100%)	BioLogic-DT (n = 5)	Mean: 2.6, median: 3, range: 1–3, max: 3	HD: at the physician’s discretion AC: received	Standard medical therapy (n = 5)	36–64	30
Demetriou (2004)	USA and Europe	FHF/SHF with gr. III-IV HE, PNF	Viral hepatitis + AO + other drug induced (49%) indeterminate (37%), PNF (14%)	HepatAssist (n = 85)	Mean: 2.9, range: 1–9		Standard medical therapy (n = 86)	10–69 (37)	70
Pollock (2004)	UK	FHF	AO (100%)	MARS (n = 6)	Max. 14		Standard medical therapy (n = 6)		
El Banayosi (2007)	Germany	ALF	Cardiogenic shock after cardiac surgery (100%)	MARS (n = 20)	Range: 1–54		Standard medical therapy (n = 20)		28
Saliba (2013)	France	ALF	AO (38%), viral hepatitis 14%) autoimmune hepatitis (12%), mushroom induced (8%), unknown (8%), drug reaction (6%), toxic agents (6%), other (9%)	MARS (n = 53)	Median: 1, range: 0–7	HD: at the physician’s discretion	Standard medical therapy (n = 49)	(40.4)	57
Larsen (2016)	Denmark, UK, Finland	ALF with gr. II or greater HE	AO (59%), unknown (21%), toxic agents (9%), viral hepatitis 6%), Budd-Chiari syndrome (1%), other (3%)	High-volume plasma exchange (n = 92)	Mean, SD: 2.4 ± 0,8, max: 3	HD: at the physician’s discretion AC: received based on local guidelines	Standard medical therapy (n = 90)	33–56	68

Table contains study characteristics of the included trials. Blank cells indicate that the data were not reported in the article. Abbreviations: ALF: acute liver failure, HE: hepatic encephalopathy, HD: hemodialysis, AC: anticoagulant, SD: standard deviation, max: maximum, USA: United States of America, FHF: fulminant hepatic failure, gr.: grade, UK: United Kingdom, AO: acetaminophen overdose, SHF: subfulminant hepatic failure, PNF: primary nonfunction following liver transplantation.

### In-hospital mortality

**Figure 2:**
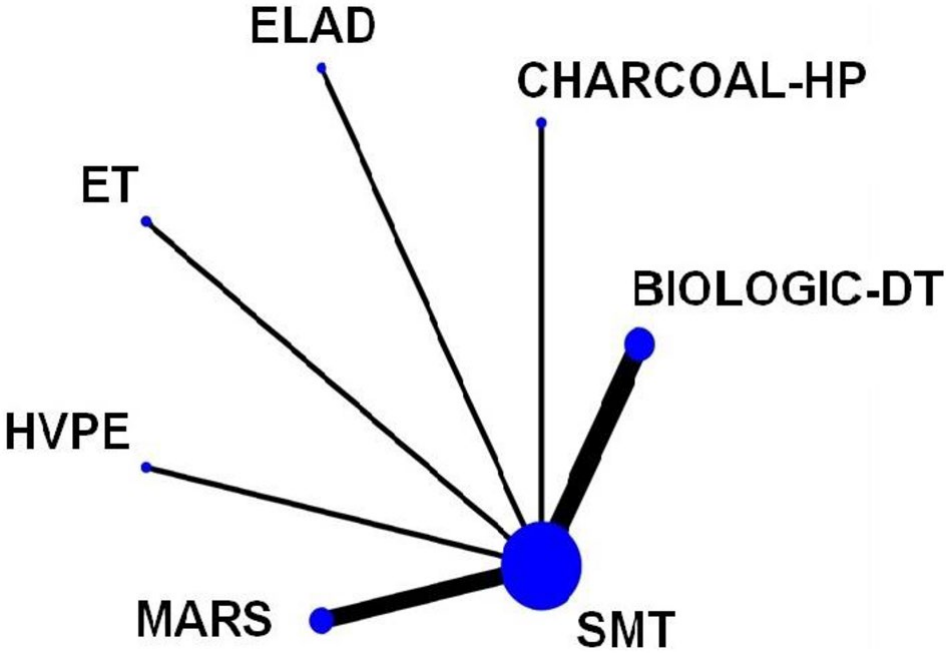
The network geometry of the eligible comparisons of in-hospital mortality. The thickness of the edges is proportional to the number of the head-to-head trials, and the size of the nodes is proportional to the number of studies in which the intervention was applied. SMT, standard medical therapy; HVPE, high-volume plasma exchange; ET, exchange transfusion; Charcoal-HP, charcoal-hemoperfusion.

The network (Fig. 2) includes eleven studies. All liver support systems were compared to standard medical therapy.

**Figure 3:**
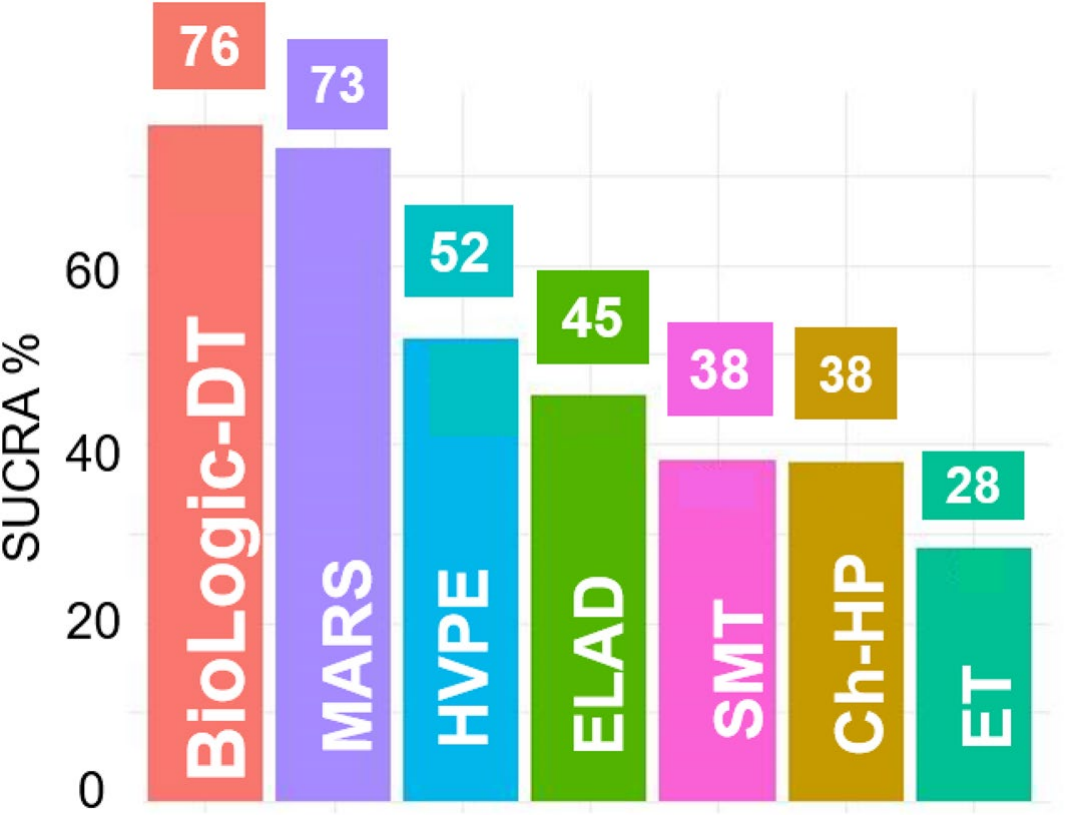
Surface under the cumulative ranking curves (SUCRA%) values of in-hospital mortality. Interventions were ranked by their posterior probability via calculating the surface under cumulative ranking (SUCRA) curve values. The higher the SUCRA value, the higher the probability for the interventions to be the best option. HVPE, high-volume plasma exchange; SMT, standard medical therapy; Ch-HP, Charcoal hemoperfusion; ET, exchange transfusion.

The SUCRA values (Fig. 3) indicate that BioLogic-DT and MARS are most likely to result in the lowest mortality. However, the results of the analysis presented in the league table (Table 2) show that there were no statistically significant differences between the interventions.

**Table 2:**
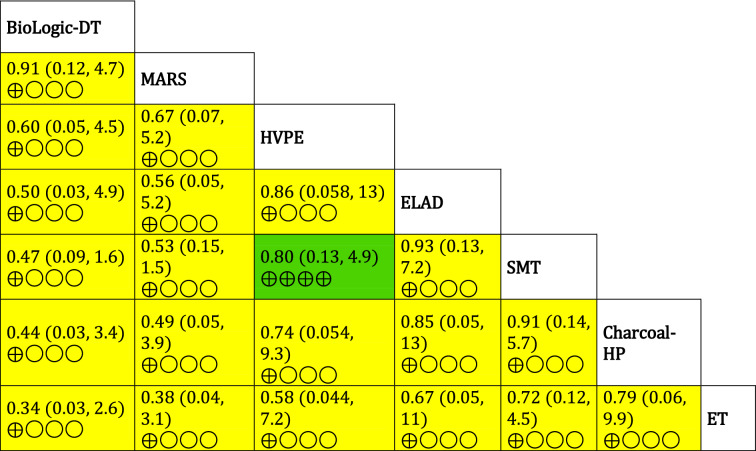
League table of pairwise comparisons regarding in-hospital mortality.

Values are given as relative risk (95% credible interval). The colour of the boxes indicates the comparisons’ overall risk of bias assessment (green: low risk of bias, yellow: some concerns, red: high risk of bias). The number of ⊕ symbols refer to the quality of evidence according to the GRADE approach (⊕ ⊕  ⊕  ⊕ high quality, ⊕  ⊕  ⊕ ◯ moderate quality, ⊕  ⊕ ◯◯ low quality, ⊕ ◯◯◯ very low quality).

### Secondary outcomes

The networks of in-hospital mortality among nonparacetamol-poisoned patients and hepatic encephalopathy are depicted in Supplementary Fig. S9 and S16.

The SUCRA values show that BioLogic-DT, charcoal hemoperfusion and MARS may be equally efficient to decrease mortality (53%, 52% and 52%, respectively) while SMT seems less effective (43%) in the nonparacetamol-poisoned patient population (Supplementary Fig. S11). Considering hepatic encephalopathy, the SUCRA rankings indicate (Supplementary Fig. S18) the ELAD therapy has the highest probability to reduce the worsening of hepatic encephalopathy while BioLogic-DT seems noticeably less appealing than SMT or ELAD (78%, 44% and 28%). On the other hand, the results from the league table (Table S1 and S2) for both outcomes confirm that no statistically significant differences can be found between the interventions.

### Long-term survival

We assessed articles in which the follow-up period was at least 30 days. In the trial of Demetriou et al. 30-day survival was 71% in the bioartificial liver-treated group (BAL) and 62% in the control group (p = 0.26, generated with Whitehead Triangular Test)^18^. Saliba et al. reported that 6-month overall survival was not significantly different in the MARS and control groups (82.1 and 75.5%, respectively, p = 0.50)^25^. Considering HVPE, Larsen et al. reported that 3-month overall survival was not improved significantly in the plasma exchange group compared to the control group, however transplant-free survival was significantly better in the HVPE-treated group after 3 months (p = 0.0058)^26^.

### Transplantation

Six trials reported on liver transplantation. Three large RCTs did not find significant differences between the control and treatment groups in the number of patients transplanted and survival rates analysing HepatAssist device, HVPE and MARS^18,25,26^. Ellis et al. examining ELAD therapy reported that 2 patients underwent transplantation and 1 survived in each group^28^. In the trial published by Wilkinson et al. 2 fulminant hepatic failure patients had liver transplantation, 1 survived and 1 underwent transplantation before the start of the trial period^20^. In the study from Mazariegos et al. 3 patients from the treatment group had liver transplantation and survived, and no patients were transplanted from control group^22^.

### Adverse events

Nine studies reported adverse events. In three trials no adverse events were observed during BioLogic-DT treatment^19–21^. With ELAD therapy tachypnoea, tachycardia, fever and bleeding occurred in two patients^28^. In a trial examining HepatAssist device thrombocytopenia was the most frequent adverse event with similar incidences between groups (33.7% vs 38.8% for controls vs interventions, respectively)^18^. During charcoal hemoperfusion renal failure, cerebral oedema and uncompensated metabolic acidosis were detected^29^. Examining HVPE, cardiac arrhythmia, acute respiratory distress syndrome (ARDS), pancreatitis, deteriorating in gas exchange, transfusion-related acute lung injury (TRALI), infections confirmed by blood culture and bleeding could be observed. The rate of adverse events were not statistically different in the treatment and control group^26^.

In a multi-center RCT MARS was tested, bleeding, death or sepsis did not occur related to MARS therapy, the majority of adverse events were related to liver transplantation and were more frequent in the not paracetamol-poisoned population^25^.

In patients with ALF due to cardiogenic shock after cardiac surgery treated with MARS no bleeding was detected due to thrombocytopenia, other adverse events were not reported^24^.

### Risk of bias and quality of evidence

Two trials were published in abstract form^22,23^. Three of the trials were adjudicated as overall low risk of bias (33%)^18,25,26^, and nine studies were judged to raise some concerns (67%) (Supplementary Fig. S22)^19–21,24,27–31^ considering mortality outcomes. Regarding hepatic encephalopathy three studies were judged to raise some concerns^19–21^ and one article was considered to be at high risk of bias^28^. Certainty of evidence for the outcomes was rated as very low for most comparisons (Supplementary, Table S3-S5).

## Discussion

The role of liver support therapies in acute liver failure is still controversial, and to the best of our knowledge, no network meta-analysis has been published in this field before. Eleven RCTs were included in the current study with mortality and hepatic encephalopathy being the patient-important outcomes. BioLogic-DT was ranked as the best treatment for in-hospital mortality and worse for hepatic encephalopathy, however this modality is not applied in clinical practice anymore. MARS therapy was the best option from the available treatments in reducing in-hospital mortality. However, with no statistically significant results, there is no solid evidence that the differences that we can see from the SUCRA values are due to chance or the interventions truly differ in their effects.

Former meta-analyses reported conflicting results considering liver support devices’ effect on mortality in acute liver failure. Zheng et al. found that bioartificial devices reduced mortality in ALF (RR: 0.69, 95% CI 0.50–0.94, P = 0.018), although from the three studies analysed two represented the same patient population^32^. Stutchfield et al. reported that based on three RCTs, liver assist devices reduced mortality (RR: 0.7, 95% CI 0.49, 1.00, P = 0.05), although the significance is not robust given the confidence interval^16^. Other previous meta-analyses did not find any significant difference between SMT and liver support techniques in the ALF population by subgroup analysis^11,14,15,33–35^.

Acetaminophen overdose is the leading cause of ALF in the USA, Australia and Europe^36–38^. Spontaneous recovery is more frequent in this patient population compared to other drug-induced, autoimmune or idiopathic ALF^36^. Therefore, emergency transplantation as a routine intervention in paracetamol poisoning has been questioned^39^. We did not have enough data in this patient population for a quantitative synthesis, however in the nonparacetamol-poisoned population no significant difference could be observed between SMT and extracorporeal liver assist devices, and the different liver support therapies applied.

Hepatic encephalopathy is an important symptom of ALF^8^. However, because of the disease’s complexity there are several different measurement scales^40^ and the result is greatly affected by the assessor^41^. Furthermore, the patients are usually sedated and mechanically ventilated, which makes the evaluation more difficult. In former meta-analyses in populations from both ACLF and ALF patients significant improvement was found in hepatic encephalopathy with ECLS systems^11,14,15,34^.

The greatest strength of this study is that the different interventions were compared to each other and were not assessed together in comparison with standard medical therapy. However, this study has certain limitations. The most important limitations are the small sample sizes, the heterogeneity of the patient populations, outcomes, and study design and the inconsistency in definitions of liver failure. We were unable to use the node-splitting analysis to examine consistency assumption because there was not enough information from the comparisons in the network. Long-term survival could not be quantitatively analysed, although it is a particularly important factor to assess the efficacy of the interventions. Finally, our network meta-analysis covers a period of more than 40 years, during which SMT has improved remarkably (that is, chronological bias).

## Conclusion

This network meta-analysis demonstrated that—as BioLogic-DT is not applied in clinical practice anymore—MARS therapy seems to be the best available option in reducing in-hospital mortality, however, no statistically significant differences could be observed among the treatments of acute liver failure considering in-hospital mortality and hepatic encephalopathy. Good-quality randomized trials are needed on currently available and new blood purification modalities to define the role of extracorporeal liver support in patients with acute liver failure.

## Methods

### Search strategy and selection criteria

The network meta-analysis was reported using the PRISMA Extension Statement for Reporting of Systematic Reviews Incorporating Network Meta-Analyses of Health Care Interventions^42^. We used the classical PICO framework for our clinical question. P: patients with acute or hyperacute liver failure (having regard to the fact that the studies were conducted in a wide range of time (1973–2016) we accepted the articles’ definition of hyperacute and acute liver failure); I and C: artificial, bioartificial liver support therapies, SMT; O: overall in-hospital mortality, mortality-by-aetiology, hepatic encephalopathy, number of patients transplanted, laboratory parameters and adverse events. Our network meta-analysis was registered with the PROSPERO registry (CRD42020160133).

For this network meta-analysis on the 4th of October 2019 we searched Medline (via PubMed), the Cochrane Central Register of Controlled Trials (CENTRAL), Web of Science, Embase and Scopus for RCTs and conference abstracts of RCTs. No restrictions were imposed on the search.

We used the following search key in all databases (complemented with the MeSH function in MEDLINE): (‘hepatic failure’ OR ‘liver failure’ OR ‘end stage liver disease’ OR cirrhosis OR 'alcoholic hepatitis') AND (‘liver support system’ OR 'liver support device' OR 'liver assist device' OR ‘artificial liver’ OR ‘bioartificial liver’ OR ‘extracorporeal liver’ OR 'albumin dialysis' OR 'extracorporeal cellular therapy' OR MARS OR Prometheus OR 'fractioned plasma separation and adsorption' OR hemadsorption OR hemoadsorption) AND random*.

Randomized controlled trials studying liver support devices in acute-on-chronic liver failure were excluded. In studies in which patients with ALF and ACLF were both involved and provided individual patient data, we only extracted the data of patients with acute liver failure. Transitivity was assessed clinically, based on the eligibility criteria of the included randomized controlled trials. As acute and hyperacute liver failure have mainly similar symptoms despite etiology, we concluded that, regarding the liver support systems’ clinical effect on these symptoms, the conditions of transitivity are satisfied.

Records from each database were downloaded into EndNote X9 citation manager (Clarivate Analytics, Philadelphia, USA) and duplicates were removed by the citation manager based on the title of the article, and then manually. The titles then the abstracts and full texts of the identified studies were screened for inclusion against the eligibility criteria by two independent review authors (KO, AK). A third party (ZM) resolved conflicts. Citing and cited articles were revised through Google Scholar, where all the additional sources were identified. The PRISMA flowchart shows the process of the article selection (Fig. 1)^43^.

### Data extraction and outcomes

All data according to study type, author and publication information, demographic data, aetiology, details of the interventions and comparators, mortality, hepatic encephalopathy, number of patients transplanted, laboratory parameters, adverse events and notes were collected in the study database (standardized template). The data from intention-to-treat analyses were extracted independently by the first (AK) and second author (KO), when conflicts arose, a third participant resolved any discrepancies (ZM).

The primary outcome of our analysis was in-hospital overall mortality. Secondary outcomes included hepatic encephalopathy (number of patients improved versus worsened plus not improved), mortality-by-aetiology, liver transplantation, long-term survival, and adverse events. We accepted the articles’ definition of adverse events. We planned to analyse changes in laboratory parameters as well but failed to do so because studies reported them in different time instants.

### Risk of bias assessment and quality of evidence

Risk of bias assessment was first performed on individual study-level according to the Revised Cochrane risk-of-bias tool for randomized trials (RoB 2)^44^. From the individual studies’ overall RoB assessment, we chose the one which was at the highest risk of bias for each intervention’s (each arm of the network) overall RoB assessment. Then we summarized the interventions’ overall RoB-assessment on the comparison level with the same method. The results of the RoB assessment are depicted in league tables. The colour of the boxes indicates the comparisons’ overall risk of bias assessment (green: low risk of bias, yellow: some concerns, red: high risk of bias). We used the Grading of Recommendations Assessment, Development, and Evaluation (GRADE) approach to assess the certainty of evidence^45^. Study limitations were evaluated based on RoB 2 tool, as detailed above. Imprecision was judged based on the sample size calculation of the article of Larsen et al.^26^. Node splitting could not be performed in any of the networks due to network geometry, consequently inconsistency could not be tested. We compared the individual studies’ populations, interventions and outcomes to rate indirectness. Publication bias was judged by the ‘comparison-adjusted’ funnel plot and Egger’s test. In the league tables we marked the quality of evidence for each comparison. Risk of bias and quality of evidence assessment were performed by two independent review authors (KO, AK), a third party (ZM) resolved conflicts.

### Statistical analysis

A Bayesian-method was used to perform pairwise meta-analyses and network meta-analysis with the random effect model. In case of missing outcome data, we replaced values with the worse outcome, i.e. in case of mortality, death, in case of hepatic encephalopathy, worsening/not improving. We used risk ratios (RR) for dichotomous data with 95% credible intervals (95% CrI). We optimized the model and generated posterior samples using the Monte-Carlo methods running in four chains. We set at least 20,000 adaptation iterations to get convergence and 10,000 simulation iterations. Network estimates (pooled direct and indirect data) of each intervention compared to standard medical therapy and to other interventions are presented in forest plots, summarized in a league table (as shown in the results section). In the network geometry the direct comparisons are presented with edges, and the thickness of the edges is proportional to the number of the head-to-head trials, and the size of the nodes is proportional to the number of studies in which the intervention was applied. We also ranked interventions by their posterior probability via calculating the SUCRA values. ‘Comparison-adjusted’ funnel plot was created with the frequentist approach, and Egger’s tests were performed in the network meta-analysis to assess small-study effect of in-hospital mortality. All calculations were performed with R (V. 3.5.2) package gemtc (V. 0.8-2) along with the Markov Chain Monte Carlo engine JAGS (V. 3.4.0) and STATA 17.0 (StataCorp LLC).

## Supplementary information

Supplementary Information.

## Data Availability

All data generated or analysed during the current study are available from the corresponding author on reasonable request.

## Abbreviations

AASLD: American Association for the Study of Liver Diseases
AC: Anticoagulant
ACLF: Acute-on-chronic liver failure
ALF: Acute liver failure
AO: Acetaminophen overdose
ARDS: Acute respiratory distress syndrome
BAL: Bioartificial liver
CrI: Credible interval
Charcoal-HP: Charcoal-hemoperfusion
EASL: European Association for the Study of the Liver
ECLS: Extracorporeal liver support
ELAD: Extracorporeal Liver Assist Device
ET: Exchange transfusion
FHF: Fulminant hepatic failure
gr: Grade
GRADE: Grading of Recommendations Assessment, Development, and Evaluation
HD: Hemodialysis
HE: Hepatic encephalopathy
HELLP-syndrome: Haemolysis, elevated liver enzymes, low platelet count
HVPE: High-volume plasma exchange
IL-6: Interleukin 6
max: Maximum
MARS: Molecular adsorbent recirculating system
PICO: P: patients I: intervention C: comparison O: outcome
PNF: Primary nonfunction following liver transplantation
PRISMA: Preferred Reporting Items for Systematic Reviews and Meta-Analyses
RCT: Randomized controlled trials
RoB2: Cochrane risk-of-bias tool for randomised trials
RR: Risk ratio
SD: Standard deviation
SHF: Subfulminant hepatic failure
SMT: Standard medical therapy
SUCRA: Surface under the cumulative ranking curves
TNF α: Tumor necrosis factor alpha
TRALI: Transfusion-related acute lung injury
UK: United Kingdom
USA: United States of America
