# Supplementary material for: Efficacy and safety of liver support devices in acute and hyperacute liver failure: a systematic review and network meta-analysis
Source: Sci Rep. 2021 Feb 18;11:4189. doi: 10.1038/s41598-021-83292-z (PMC7893063; doi:10.1038/s41598-021-83292-z)

Supplementary material

Efficacy and safety of liver support devices' in acute and hyperacute liver failure: A systematic review and network meta-analysis

Anna Kanjo[1,2,3], Klementina Ocskay[1], Noémi Gede[1], Szabolcs Kiss[1,3,] Zsolt Szakács[1,4], Andrea Párniczky[1,2,3], Steffen Mitzner[5], Jan Stange[5], Péter Hegyi[1,3,4], Zsolt Molnár*[1,3, 6]

[1] Institute for Translational Medicine, Medical School, University of Pécs, Pécs, Hungary

[2] Heim Pál National Paediatric Institute, Budapest, Hungary

[3] Doctoral School of Clinical Medicine, University of Szeged, Szeged, Hungary

[4] János Szentágothai Research Centre, University of Pécs, Pécs, Hungary

[5] Division of Nephrology, Department of Medicine, University of Rostock, Rostock, Germany

[6] Department of Anesthesiology and Intensive Therapy, Poznan University for Medical Sciences, Medical Faculty, Poznan, Poland

Table of contents

1. In-hospital mortality 3

Figure S1 3

Figure S2 3

Figure S3 3

Figure S4 4

Figure S5 4

Figure S6 4

Figure S7 5

Figure S8 5

2. In-hospital mortality in nonparacetamol-poisoned patients 6

Figure S9 6

Figure S10 6

Figure S11 7

Table S1 7

Figure S12 8

Figure S13 8

Figure S14 8

Figure S15 8

3. Hepatic encephalopathy 9

Figure S16 9
Figure S17 9
Figure S18 10
Table S2 10
Figure S19 11
Figure S20 11
Figure S21 11
4. Risk of bias assessment 12
Figure S22 12
Figure S23 13
Figure S24 13
5. Quality of evidence 13
Table S3 15
Table S4 17
Table S5 18
Figure S25 19

1. **In-hospital mortality**

Figure S1. Forest plot for in-hospital mortality, interventions compared to SMT

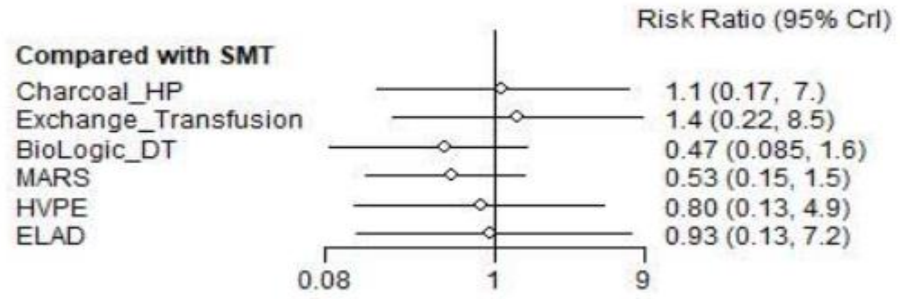

Abbreviations: SMT: standard medical therapy; Charcoal-HP: charcoal-hemoperfusion; HVPE: high-volume plasma exchange

Figure S2. Forest plot for in-hospital mortality, interventions compared to HVPE

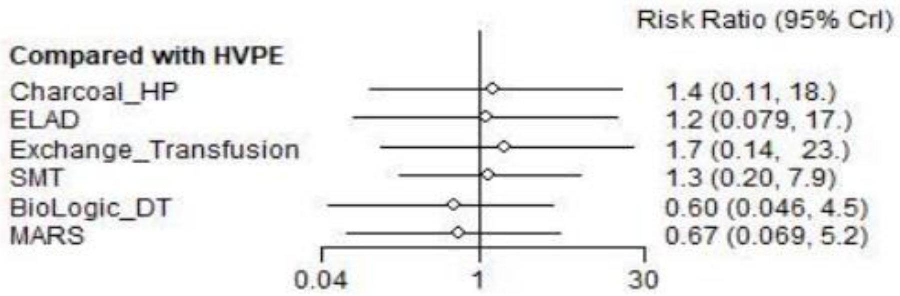

Abbreviations: HVPE: high-volume plasma exchange; SMT: standard medical therapy; Charcoal-HP: charcoal-hemoperfusion

Figure S3. Forest plot for in-hospital mortality, interventions compared to ELAD

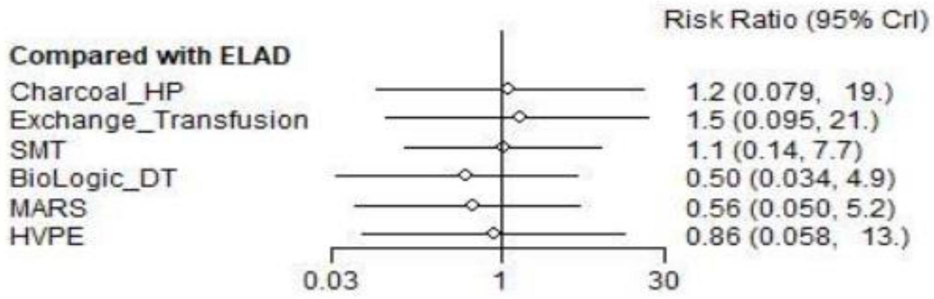

Abbreviations: HVPE: high-volume plasma exchange; SMT: standard medical therapy; Charcoal-HP: charcoal-hemoperfusion

Figure S4. Forest plot for in-hospital mortality, interventions compared to charcoal-hemoperfusion

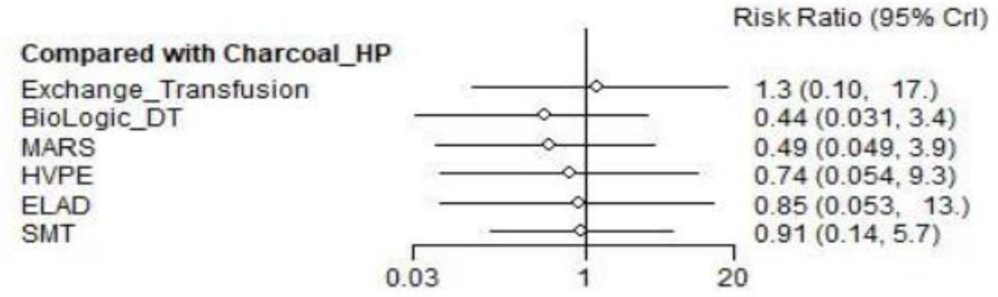

Abbreviations: HVPE: high-volume plasma exchange; SMT: standard medical therapy; Charcoal-HP: charcoal-hemoperfusion

Figure S5. Forest plot for in-hospital mortality, interventions compared to exchange-transfusion

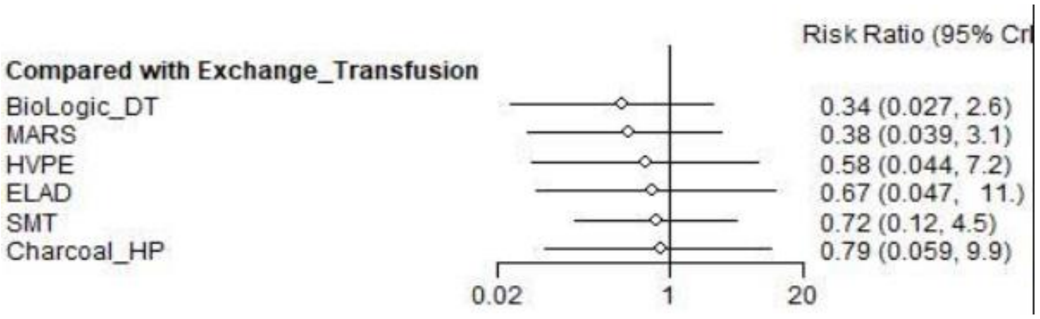

Abbreviations: HVPE: high-volume plasma exchange; SMT: standard medical therapy; Charcoal-HP: charcoal-hemoperfusion

Figure S6. Forest plot for in-hospital mortality, interventions compared to BioLogic-DT

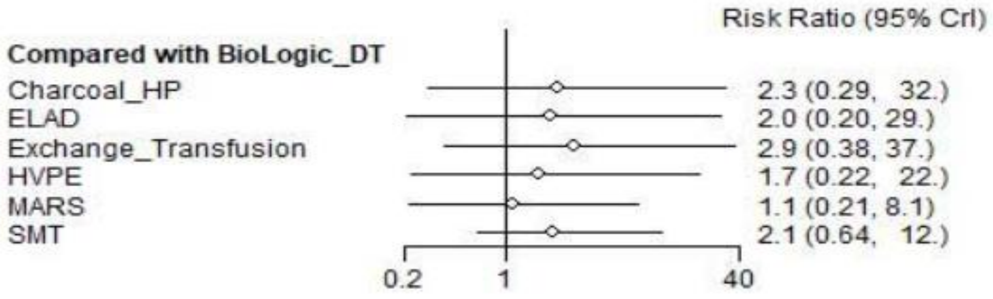

Abbreviations: HVPE: high-volume plasma exchange; SMT: standard medical therapy; Charcoal-HP: charcoal-hemoperfusion

Figure S7. Forest plot for in-hospital mortality, interventions compared to MARS

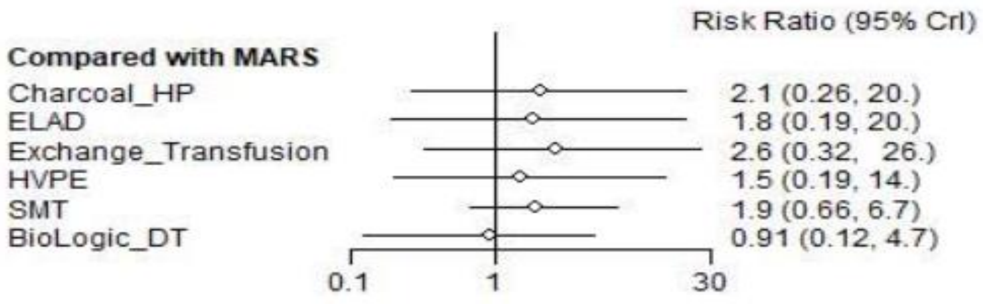

Abbreviations: HVPE: high-volume plasma exchange; SMT: standard medical therapy; Charcoal-HP: charcoal-hemoperfusion

Figure S8. Cumulative ranking curves of in-hospital mortality

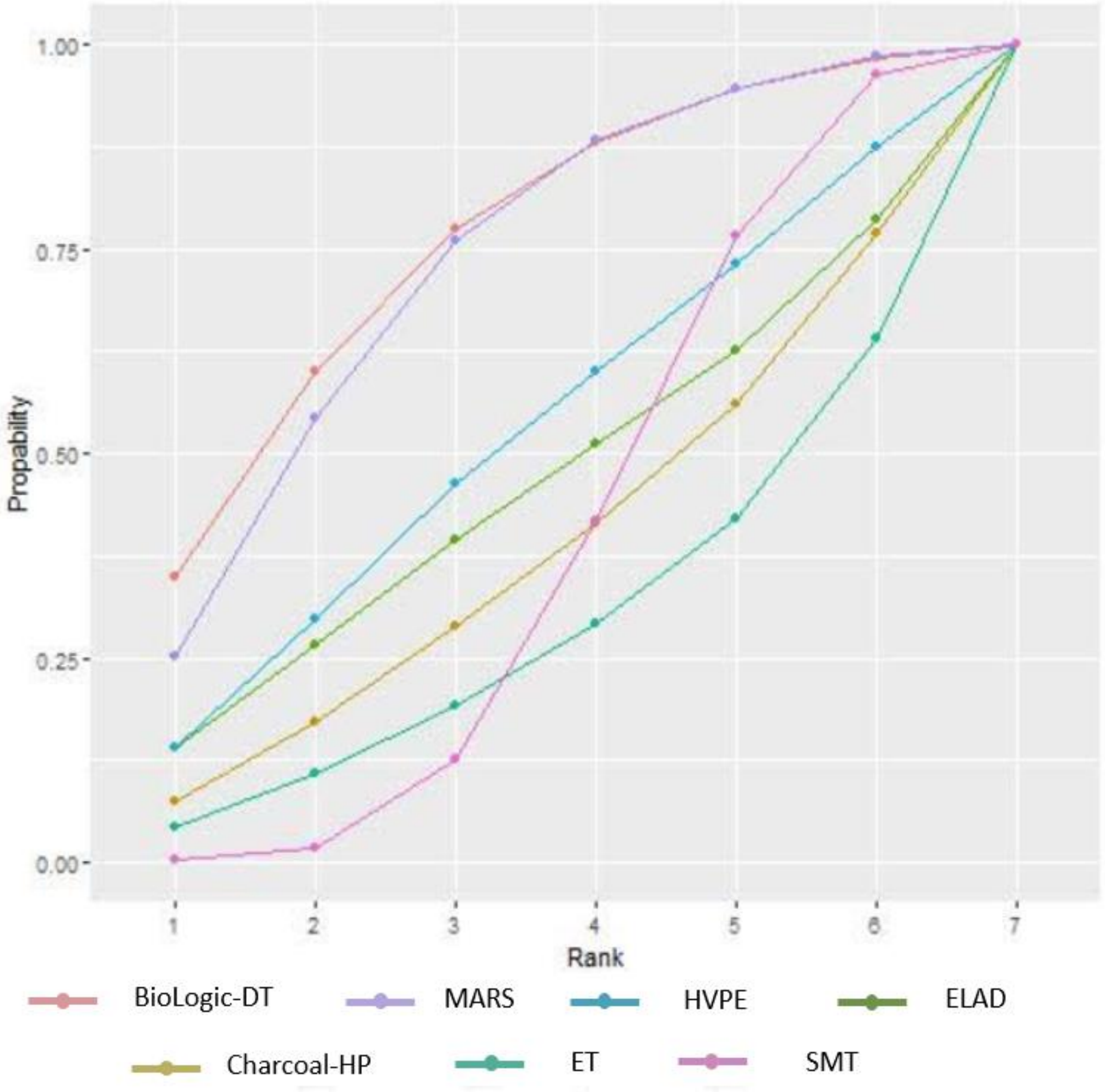

Abbreviations: HVPE: high-volume plasma exchange; Charcoal-HP: charcoal-hemoperfusion ET: exchange transfusion; SMT: standard medical therapy

2. **In-hospital mortality in nonparacetamol-poisoned patients**

Figure S9. The network geometry of the eligible comparisons of in-hospital mortality in nonparacetamol-poisoned patients.

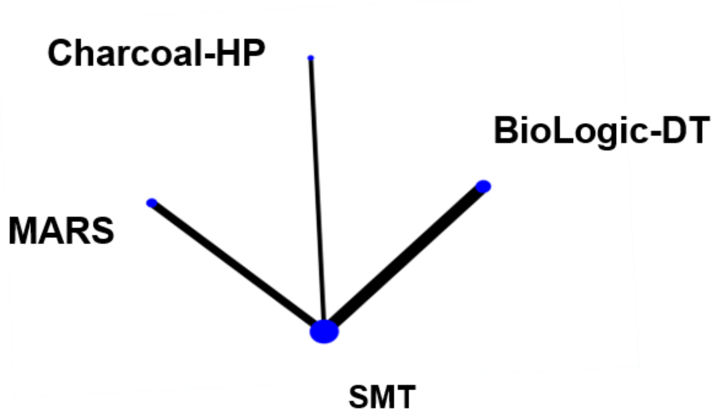

The thickness of the edges is proportional to the number of the head-to-head trials, and the size of the nodes is proportional to the number of studies in which the intervention was applied. Abbreviations: SMT: standard medical therapy, Charcoal-HP: charcoal-hemoperfusion

Figure S10. Cumulative ranking curves of in-hospital mortality in nonparacetamol-poisoned patients

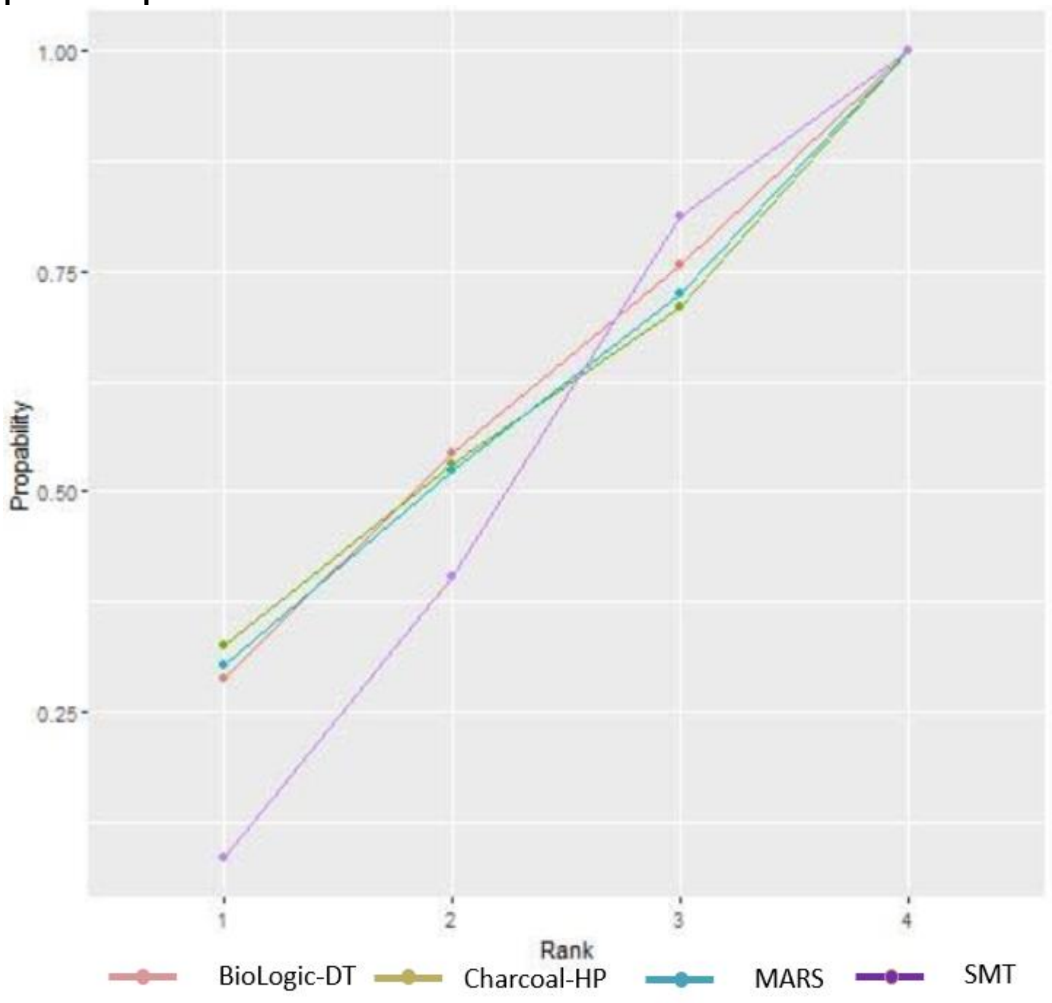

Abbreviations: SMT: standard medical therapy, Charcoal-HP: charcoal-hemoperfusion

Figure S11. Surface under the cumulative ranking curves (SUCRA%) values of in-hospital mortality in nonparacetamol-poisoned patients.

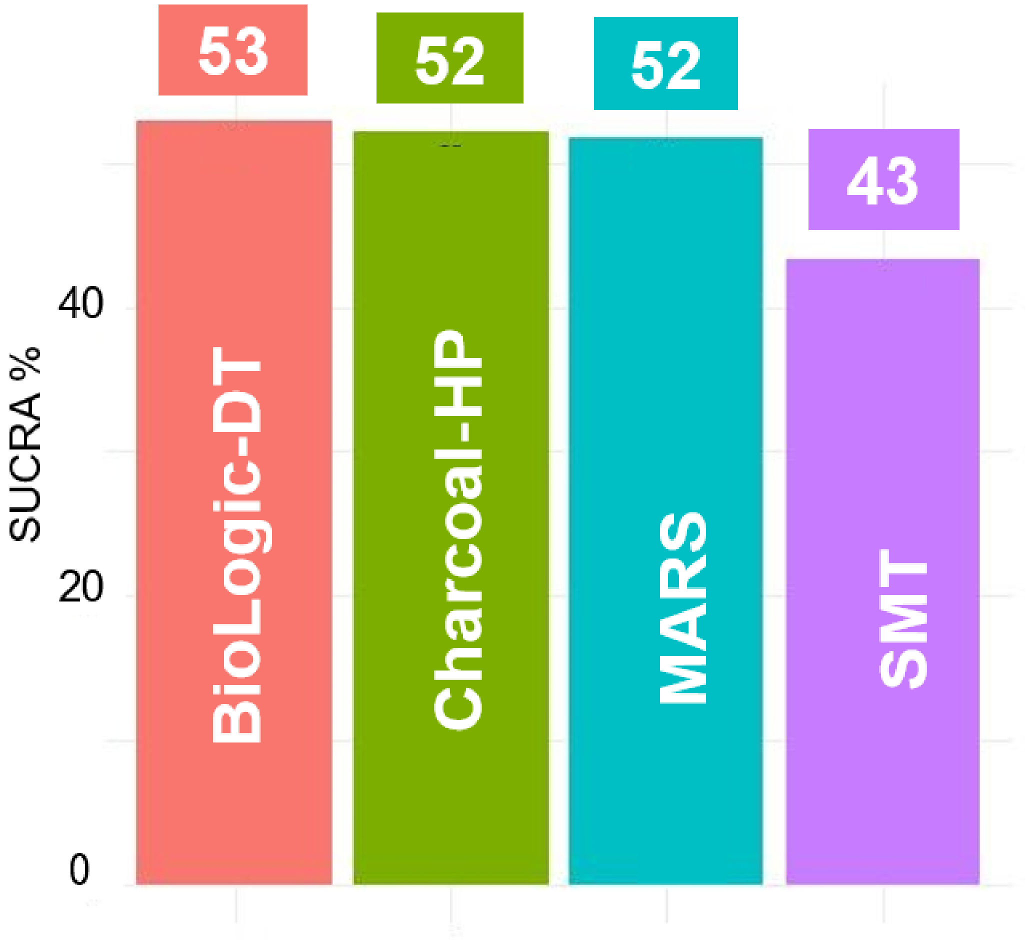

Abbreviations: Charcoal-HP: charcoal-hemoperfusion; SMT: standard medical therapy

Table S1. League table of in-hospital mortality of non-paracetamol poisoned patients

**BioLogic-DT**			
1.0 (0.17, 4.4)	**Charcoal-HP**		
0.99 (0.20, 3.7)	0.99 (0.16, 5.5)	**MARS**	
0. 93 (0.32, 2.1)	0. 94 (0.23, 3.6)	0.94 (0.32, 2.9)	**SMT**

The league table contains the risk ratios /RR/ (credible intervals /CrI/) for every possible comparison of the interventions. All the comparisons' overall risk of bias assessments were judged to raise some concern and according to the GRADE approach all comparisons were judged as very low quality ⊕◯◯◯.

Abbreviations: Charcoal-HP: charcoal-hemoperfusion; SMT: standard medical therapy

Figure S12. Forest plot for in-hospital mortality in nonparacetamol-poisoned patients, interventions compared to charcoal-hemoperfusion

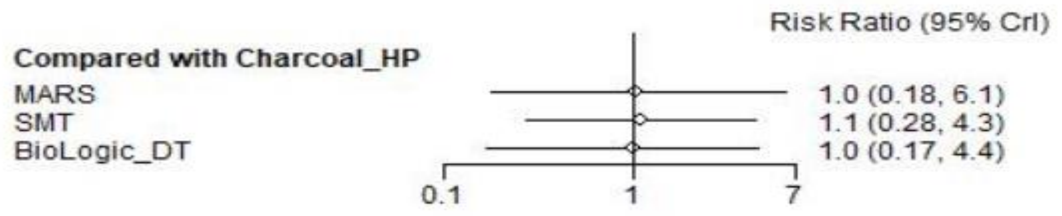

Abbreviations: Charcoal-HP: charcoal-hemoperfusion; SMT: standard medical therapy

Figure S13. Forest plot for in-hospital mortality in nonparacetamol-poisoned patients, interventions compared to MARS

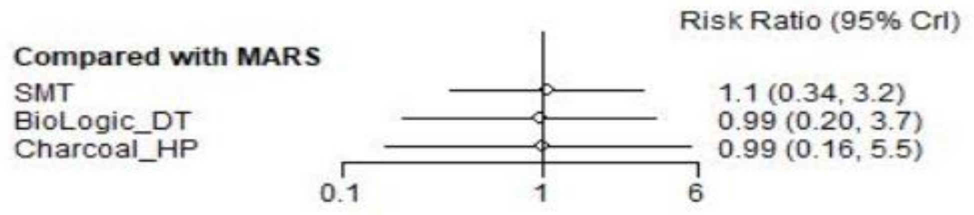

Abbreviations: Charcoal-HP: charcoal-hemoperfusion; SMT: standard medical therapy

Figure S14. Forest plot for in-hospital mortality in nonparacetamol-poisoned patients, interventions compared to BioLogic-DT

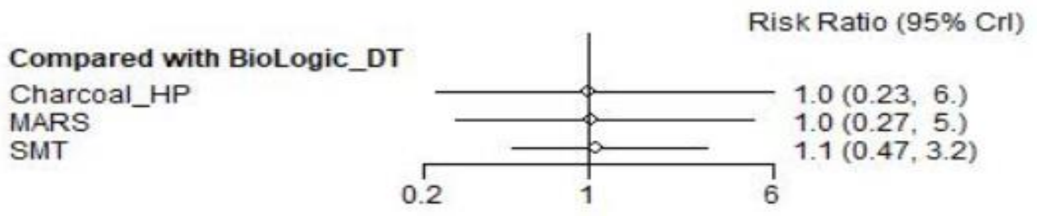

Abbreviations: Charcoal-HP: charcoal-hemoperfusion; SMT: standard medical therapy

Figure S15. Forest plot for in-hospital mortality in nonparacetamol-poisoned patients, interventions compared to standard medical therapy

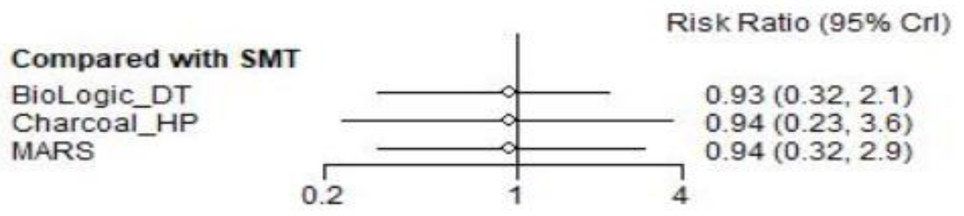

Abbreviations: Charcoal-HP: charcoal-hemoperfusion; SMT: standard medical therapy

3. **Hepatic encephalopathy**

Figure S16. The network geometry of the eligible comparisons of in-hospital mortality in nonparacetamol-poisoned patients.

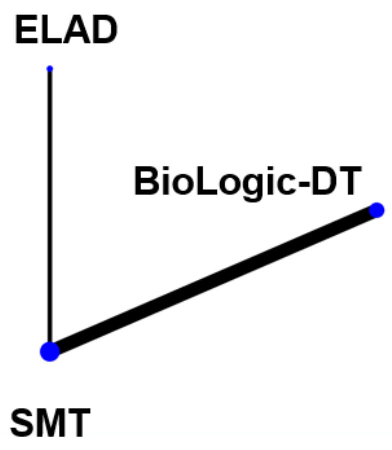

Abbreviation: SMT: standard medical therapy

Figure S17. Cumulative ranking curves of hepatic encephalopathy

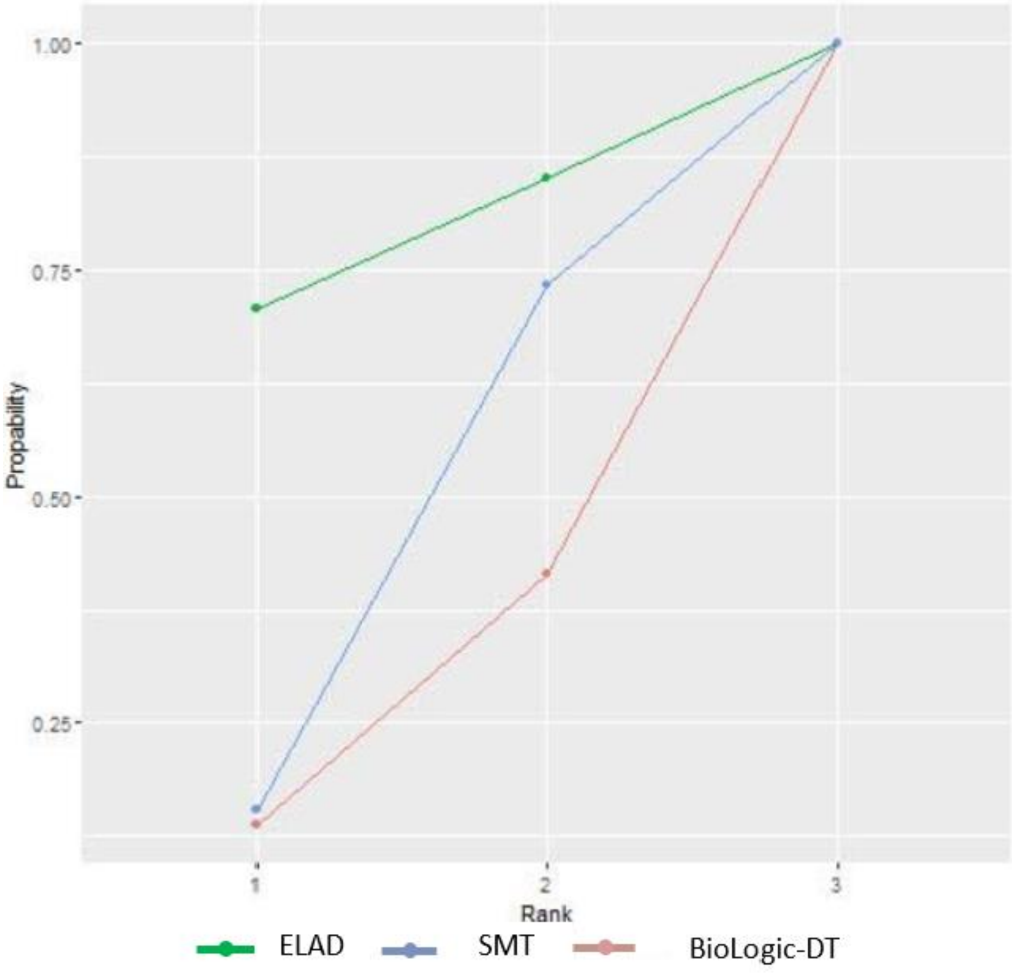

Abbreviation: SMT: standard medical therapy

Figure S18. Surface under the cumulative ranking curves (SUCRA%) values of hepatic encephalopathy

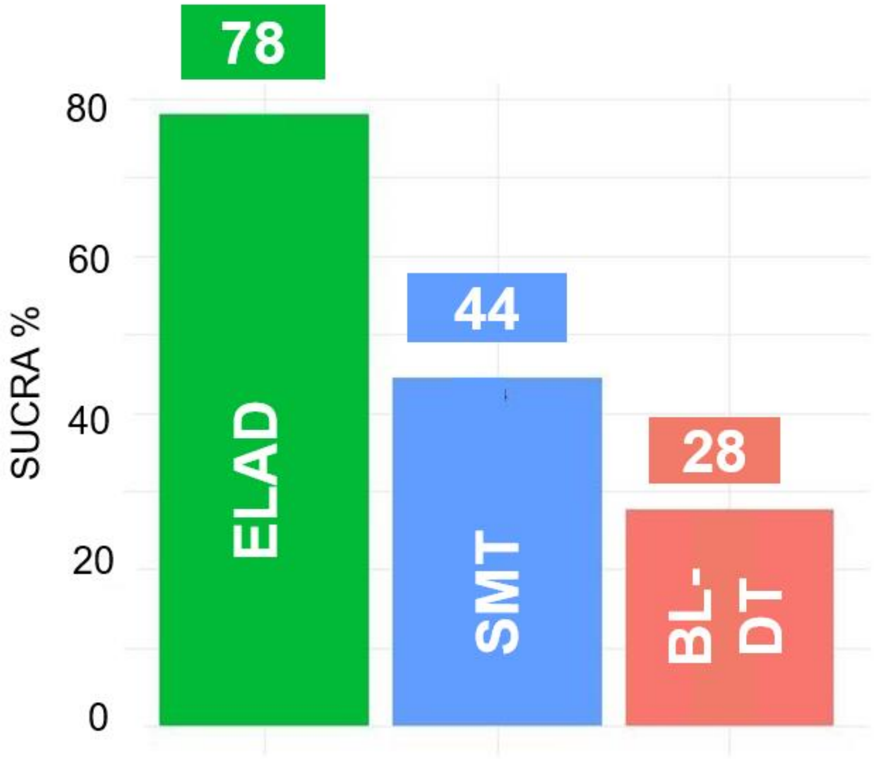

Abbreviations: SMT: standard medical therapy; BL-DT: BioLogic-DT

Table S2. League table of hepatic encephalopathy

**ELAD**		
**0.65 (0.17-2.1)**	**SMT**	
**0.56 (0.12-2.3)**	**0.85 (0.37-2)**	**BioLogic-DT**

The league table contains the risk ratios /RR/ (credible intervals /CrI/) for every possible comparison of the interventions. The event was the number of patients whose hepatic encephalopathy worsened/not improved. The colour of the boxes indicates the comparisons' overall risk of bias assessment (green: low risk of bias, yellow: some concerns, red: high risk of bias). According to the GRADE approach all comparisons were judged as very low quality ⨁◯◯◯.

Figure S19. Forest plot hepatic encephalopathy, interventions compared to standard

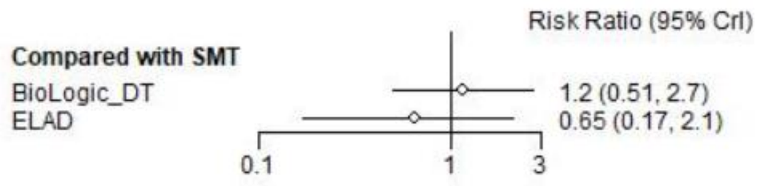

medical therapy

Abbreviations: SMT: standard medical therapy

Figure S20. Forest plot hepatic encephalopathy, interventions compared to BioLogic-DT

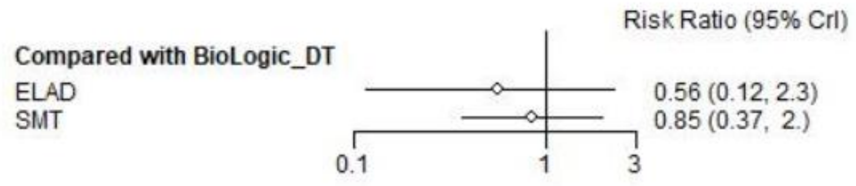

Abbreviations: SMT: standard medical therapy

Figure S21. Forest plot hepatic encephalopathy, interventions compared to ELAD

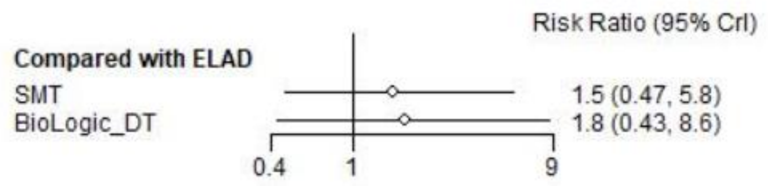

Abbreviations: SMT: standard medical therapy

4. Risk of bias assessment

Figure S22. Risk of bias assessment

Studies with intention-to-treat

Unique ID	Study ID	Experimental	Comparator	Outcome	Weight	Randomization process	Deviations from intended interven	Missing outcome data	Measurement of the outcome	Selection of the reported result	Overall
Mazariegos_1997	Demetriou_2004	BioLogic-DT	SMT	in-hospital mortality		+	?	+	+	?	!
O'Grady_1988		Charcoal hemope	SMT	in-hospital mortality		?	+	+	+	?	!
Hughes_1994		BioLogic-DT	SMT	in-hospital mortality		+	+	+	+	?	!
Wilkinson_1998		BioLogic-DT	SMT	in-hospital mortality		?	+	+	+	?	!
Polllock_2004		MARS	SMT	in-hospital mortality		+	+	+	+	?	!
Redeker_1973		Exchange transfu	SMT	in-hospital mortality		+	+	+	+	?	!
Larsen_2016		High-volume plasi	SMT	in-hospital mortality		+	+	+	+	+	+
Saliba_2013		MARS	SMT	in-hospital mortality		+	+	+	+	+	+
Elllis_1996		ELAD	SMT	in-hospital mortality		+	+	+	+	?	!
Elllis_1999		BioLogic-DT	SMT	in-hospital mortality		+	+	+	+	?	!
El Banayosi_2007		MARS	SMT	in-hospital mortality		+	+	+	+	?	!
Demetriou_2004		HepatAssist	SMT	thirty-day survival		+	+	+	+	+	+
Hughes_1994_HE		BioLogic-DT	SMT	hepatic encephalopathy		+	+	+	?	?	!
Elllis_1996_HE		ELAD	SMT	hepatic encephalopathy		+	+	+	−	?	−
Ellis_1999_HE		BioLogic-DT	SMT	hepatic encephalopathy		+	+	+	+	?	!
Wilkinson_1998_HE		BioLogic-DT	SMT	hepatic encephalopathy		?	+	+	+	?	!

+ Low risk
? Some concerns
− High risk

Figure S23. Risk of bias assessment of mortality outcomes, broken down to tools, shown in percentage

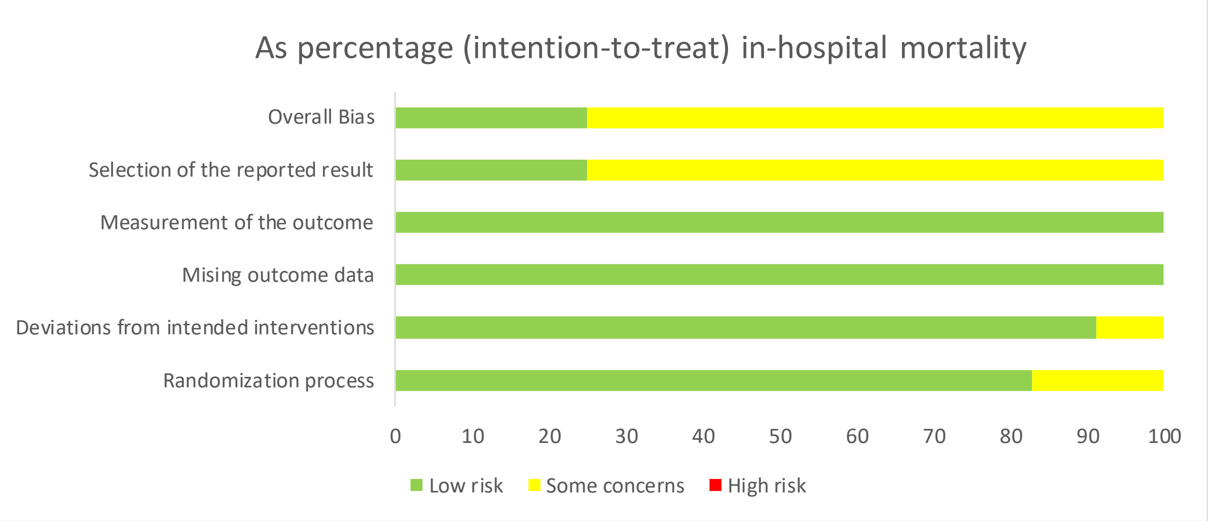

Figure S24. Risk of bias assessment of hepatic encephalopathy, broken down to tools, shown in percentage

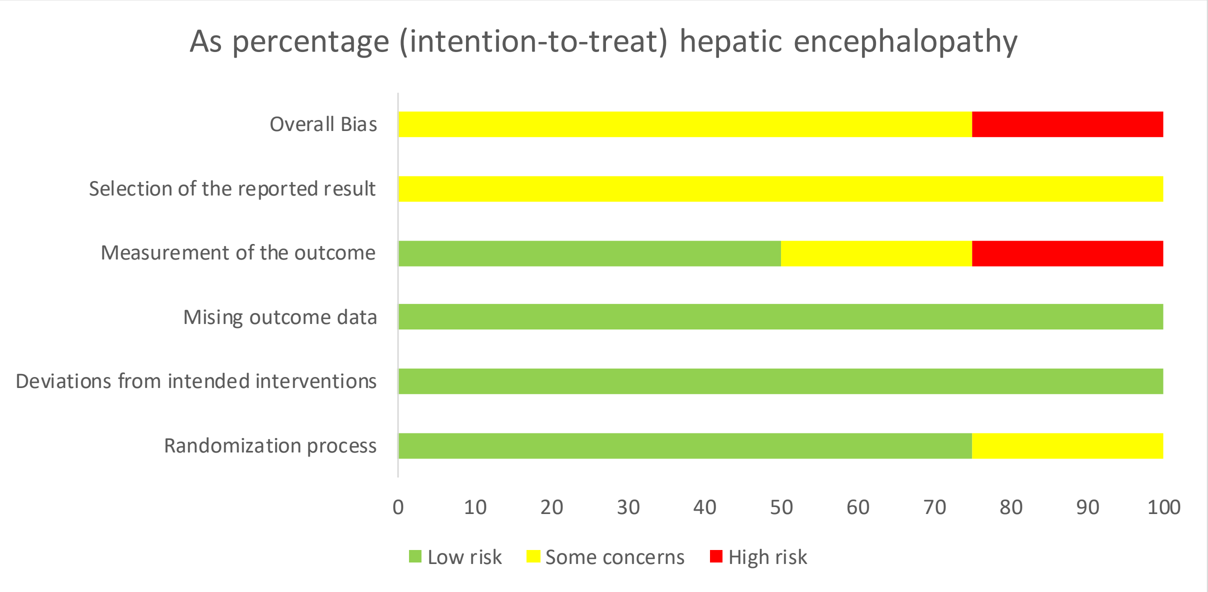

5. Quality of evidence

Risk of bias assessment was first performed on individual study-level according to the Revised Cochrane risk-of-bias tool for randomized trials (RoB 2). From the individual studies we chose the one which was at the highest risk of bias. Then we summarized the interventions' overall RoB-assessment on the comparison level with the same method. When the comparison was at high risk of bias, we downgraded the quality of evidence by two, if it was judged to raise some concerns, we downgraded the quality of evidence by one.

Imprecision was judged based on the sample size calculation of the article of Larsen, 2016. Except for that study, none of the articles had the appropriate number of patients, thus we downgraded the quality of evidence in each comparison in every outcome by two.

Node splitting could not be performed due to the geometry of the networks, as a result inconsistency could not be tested.

Indirectness: the study populations were heterogenous in most of the studies, with different etiologies and disease onset. Methodological differences were found among the studies according to renal replacement and anticoagulant therapy detailed in *Table 1, Ancillary hemodialysis and use of anticoagulation therapy*. Differences in outcome measures were found concerning hepatic encephalopathy, according to the different scores applied. Indirectness could not be measured where there was only one head-to-head trial between two interventions.

'Comparison-adjusted' funnel plot was created with the frequentist approach, and Egger's test were performed in a network meta-analysis to assess small-study effect of in-hospital mortality. Asymmetry was not significant thus downgrading was not necessary. Considering in-hospital mortality in nonparacetamol-poisoned patients and hepatic encephalopathy due to the low number of articles funnel plot and Egger's test could not be performed.

The quality of evidence firstly was judged where head-to-head trials exist, then we chose the lowest quality of evidence for the indirect comparisons.

Table S3 Summary of findings table of in-hospital mortality

	BioLogic-DT vs SMT	MARS vs SMT	HVPE vs SMT	ELAD vs SMT	Charcoal-HP vs SMT	ET vs SMT
**Study limitations**[1]	↓	↓	-	↓	↓	↓
**Comments**	some concerns	some concerns	low risk of bias	some concerns	some concerns	some concerns
**Imprecision**[2]	↓↓	↓↓	-	↓↓	↓↓	↓↓
**Inconsistency**[3]	-	-	-	-	-	-
**Indirectness**[4]	↓	↓	-	-	-	-
**Comments**	different study populations, HD was performed at the physician's discretion (Ellis, 1999) or was not allowed (Wilkinson, 1998; Hughes 1994)	different study populations, HD was performed at the physician's discretion				
**Publication bias**[5]	-	-	-	-	-	-
**GRADE**	very low quality ⊕◯◯◯	very low quality ⊕◯◯◯	high quality ⊕⊕⊕⊕	very low quality ⊕◯◯◯	very low quality ⊕◯◯◯	very low quality ⊕◯◯◯

The table includes information from 11 studies and 479 patients

[1] Detailed information on study limitations can be found in *Figure S22-24*

[2] Imprecision was judged based on the sample size calculation of the article of Larsen, 2016.
[3] Node splitting could not be performed due to network geometry, inconsistency could not be tested.
[4] Indirectness could not be judged where there was only one head-to-head trial between two interventions
[5] Publication bias was judged by the ‘comparison-adjusted’ funnel plot and Egger’s test (*Figure S25*), asymmetry is not significant thus downgrading was not necessary

Table S4 Summary of findings table of in-hospital mortality in nonparacetamol-poisoned patients

	BioLogic-DT vs SMT	MARS vs SMT	Charcoal-HP vs SMT
**Study limitations**[1]	↓	↓	↓
**Comments**	some concerns	some concerns	some concerns
**Imprecision**[2]	↓↓	↓↓	↓↓
**Inconsistency**[3]	-	-	-
**Indirectness**[4]	↓	↓	-
**Comments**	different study populations, HD was performed at the physician's discretion (Ellis, 1999) or was not allowed (Wilkinson, 1998; Hughes 1994)	different study populations, HD was performed at the physician's discretion	
**Publication bias**[5]	-	-	-
**GRADE**	very low quality ⨁◯◯◯	very low quality ⨁◯◯◯	very low quality ⨁◯◯◯

The table includes information from 6 studies and 150 patients

[1] Detailed information on study limitations can be found in Figure S22-24

[2] Imprecision was judged based on the sample size calculation of the article of Larsen, 2016.

[3] Node splitting could not be performed due to network geometry, inconsistency could not be tested.

[4] Indirectness could not be judged where there was only one head-to-head trial between two interventions

[5] Due to the low number of articles funnel plot and Egger's test could not be performed

Table S5 Summary of findings table of hepatic encephalopathy

	BioLogic-DT vs SMT	ELAD vs SMT
**Study limitations[1]**	↓	↓↓
**Comments**	some concerns	high risk of bias
**Imprecision[2]**	↓↓	↓↓
**Inconsistency[3]**	-	-
**Indirectness[4]**	↓	-
**Comments**	different applied neurological tests/scales, no detailed information on the implementation, the result is greatly affected by the assessor	
**Publication bias**	-	-
**GRADE**	very low quality ⊕◯◯◯	very low quality ⊕◯◯◯

The table includes information from 4 studies and 47 patients

[1] Detailed information on study limitations can be found in Figure S22-24

[2] Imprecision was judged based on the sample size calculation of the article of Larsen, 2016.

[3] Node splitting could not be performed due to network geometry, inconsistency could not be tested.

[4] Indirectness could not be judged where there was only one head-to-head trial between two interventions

[5] Due to the low number of articles funnel plot and Egger's test could not be performed

Figure S25 'Comparison-adjusted' Funnel plot and Egger's test of in-hospital mortality

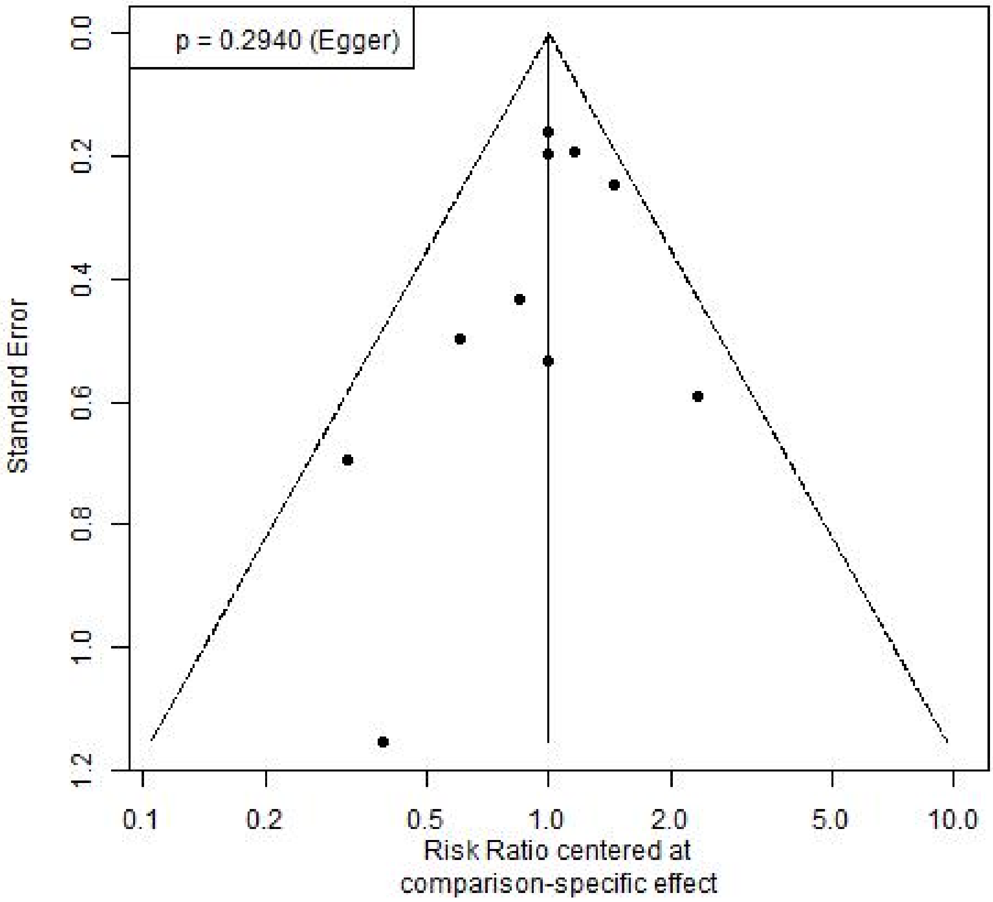

Supplement: Supplementary file 1 — Supplementary Information. [file 41598_2021_83292_MOESM1_ESM.pdf]
